# Supplementary material for: Comprehensive Elemental Profiling of Romanian Honey: Exploring Regional Variance, Honey Types, and Analyzed Metals for Sustainable Apicultural and Environmental Practices
Source: Foods. 2024 Apr 19;13(8):1253. doi: 10.3390/foods13081253 (PMC11048993; doi:10.3390/foods13081253)
Supplement: Supplementary file 1 [file foods-13-01253-s001.zip › foods-2936337-supplementary.pdf]

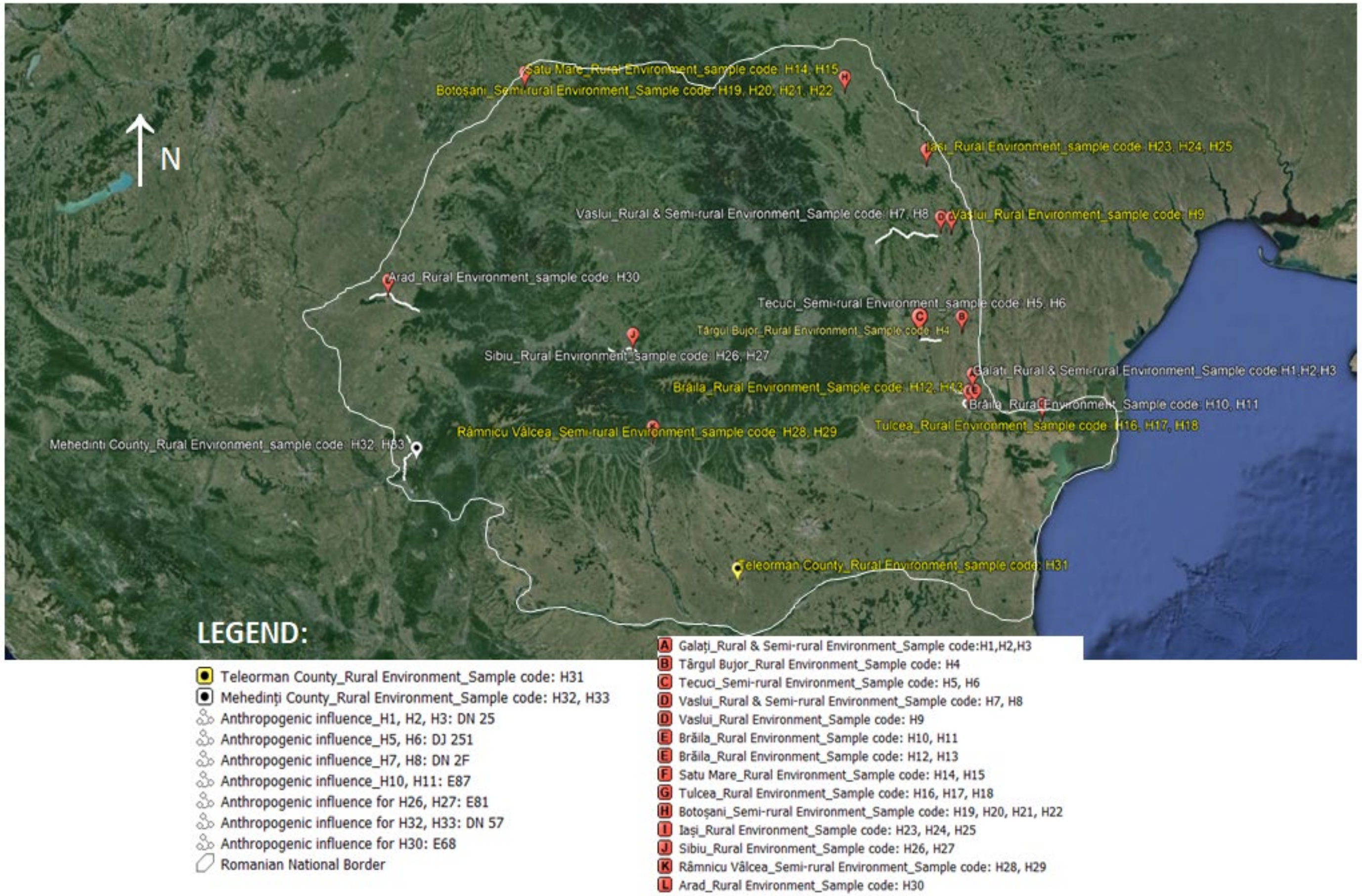

Figure S1. Geographic distribution of honey samples and identified pollutions sources

Table S1. Macroelements and microelements concentrations reported in various studies from honey.

No of Items	Denomination	Country	Location	No. of samples	Method	Macroelements				Microelements: Trace Elements							Ref.
						^{19}K	^{23}Na	^{24}Mg	^{43}Ca	^{7}Li	^{27}Al	^{56}Fe	^{64}Cu	^{65}Zn	^{88}Sr	^{9}Be	
1.	Acacia	Romania	Domasnea	3	AAS ppm	95.95	26.062	20.350	185.80	–	–	2.7955	0.1435	< 0.125	–	–	[32]
			Farliug			116.00	28.080	21.260	197.10			3.7795	0.1885	0.2305			
			Bala			100.90	27.165	21.005	195.05			4.8055	0.0955	0.4200			
2.	Acacia, Lime, sunflower, and polyfloral honey		Dolj Mehedinți Gorj	–	AAS ppm	356-735	1.94-51.06	7.05-23.07	5.8-76.46	–	–	1.13-7.34	0.15-0.56	< 0.03-1.86	–	–	[33]
3.	Polyfloral		Copșa Mică	–	FAAS GFAAS mg/kg	–	–	–	–	–	–	–	2.00-33.00	15.00-36.40	–	–	[34]
4.	Mixed Flowers P1		Timiș	–	AAS ppm	–	–	–	–	–	–	10.49	18.89	0.978	–	–	[35]
	Mixed Flowers P0					–	–	–	–	–	–	17	39.55	-	–	–	
	Linden P1					–	–	–	–	–	–	80.32	35.543	-	–	–	
	Linden P0					–	–	–	–	–	–	67.89	75.5	0.1477	–	–	
	Rape					–	–	–	–	–	–	47.24	48.17	0.336	–	–	
	Acacia					–	–	–	–	–	–	23.18	10.73	1.48	–	–	
5.	Knotweed		Banat	10	AAS mg/kg	81.332	7.673	35.280	32.521	–	–	4.261	4.272	3.133	–	–	[36]
	Linden					85.706	12.510	40.700	70.547	–	–	8.457	5.139	3.881	–	–	
	Acacia					56.749	13.025	35.179	37.370	–	–	7.284	6.986	4.550	–	–	
	Oilseed rape					82.367	7.571	39.846	67.473	–	–	6.237	4.505	2.780	–	–	
	Sunflower					65.089	8.203	38.097	54.280	–	–	7.218	5.037	3.177	–	–	
6.	Acacia		Bihor	6	ICP-OES mg/kg	213.522	–	3.128	45.160	–	0.049	0.396	0.347	0.371	–	–	[37]
	Honeydew			18		1201.296	–	46.392	523.940	–	1.197	1.923	0.655	2.158	–	–	
	Heather			6		1680.685	–	47.449	284.473	–	2.696	1.594	0.866	3.141	–	–	
	Lime			6		507.152	–	23.619	501.040	–	0.366	0.598	0.493	1.952	–	–	
7.	Polyfloral		Baia Mare	–	FAAS mg/kg	–	–	–	–	–	–	–	0.20-0.32	0.89-1.39	–	–	[38]
8.	Multifloral	Italy	Lazio	40	ICP-OES µg/g	237-6520	4.8-176	6.2-148	< 43-283	1.1-24	< 0.3-9.2	< 1-4.4	< 0.06-5.4	< 0.5-8.9	0.04-2.8	0.06-1.1	[39]

9.	Unifloral	Bulgaria	–	200	ICP-AES mg/kg	126-1628	7.22-16.3	4.8-97	32-110	–	0.35-1.58	0.35-4.37	< 0.01-0.45	0.08-1.04	0.11-0.40	–	[40]
10.	Polyfloral	France	-	86	ICP-AES ppm	–	–	1.43-109.50	2.98-108.50	0.02-0.24	0.05-1.44	0.13-10	0.03-2.30	0.04-5.96	–	–	[41]
11.	Fir	Greek	Messinia	207	ICP-OES mg/kg	–	–	79.05	25.79	–	23.78	4.03	0.77	1.04	–	0.79	[42]
12.	Lavender	Algeria	Sidi Djillali	37	AAS (mg/kg)	808.00	21.60	142.00	56.20	–	–	59.60	3.66	2.62	–	–	[43]
	Rosemary					460.00	49.20	126.00	58.50	–	–	24.50	5.70	2.39	–	–	
	Multifloral					418.00	37.00	142.00	64.90	–	–	24.40	4.46	4.41	–	–	
13.	Acacia	Hungary	–	187	MP-AES (mg/kg)	327.9	23.5	10.4	28.1	–	1.6	–	–	2.6	–	–	[44]
	Honeydew					2069.1	62.2	118.7	134.4	–	1.0	–	–	2.5	–	–	
	Forest					1892.7	46.1	71.5	121.5	–	1.8	–	–	4.2	–	–	
	Sunflower					217.7	55.1	49.7	217.7	–	11.5	–	–	5.5	–	–	
	Chestnut					2466.3	43.2	52.3	161.4	–	1.5	–	–	1.2	–	–	
	Rape					399.4	22.8	19.2	68.6	–	2.2	–	–	2.3	–	–	
	Multifloral					696.5	17.4	29.5	110.9	–	1.4	–	–	3.1	–	–	

Table S2. Microelements concentrations reported in various studies from honey.

No of Items	Denomination	Country	Location	No. of samples	Method	Microelements: Trace Elements														Ref.
						^{51}V	^{52}Cr	^{55}Mn	^{59}Co	^{60}Ni	^{70}Ga	^{79}Se	^{85}Rb	^{208}Ag	^{204}Tl	^{209}Bi	^{115}In	^{133}Cs	^{137}Ba	
1.	Acacia	Romania	Domasnea	3	AAS ppm	–	ND	0.2635	–	–	–	–	–	–	–	–	–	–	–	[32]
			Farliug				ND	0.5515												
			Bala				ND	0.3855												
2.	Acacia, Lime, sunflower, and polyfloral honey		Dolj Mehedinți Gorj	–	AAS ppm	–	–	0.10	–	0.003-0.025	–	–	–	–	–	–	–	–	–	[33]

3.	Polyfloral		Copșa Mică	–	FAAS GFAAS mg/kg	–	–	–	–	–	–	–	–	–	–	–	–	–	–	[34]
4.	Mixed Flowers P1		Timiș	–	AAS ppm	–	–	0.512	–	–	–	–	–	–	–	–	–	–	–	[35]
	Mixed Flowers P0					–	–	0.044	–	–	–	–	–	–	–	–	–	–	–	
	Linden P1					–	10.34	2.526	–	7.64	–	–	–	–	–	–	–	–	–	
	Linden P0					–	6.875	4.803	–	0.664	–	–	–	–	–	–	–	–	–	
	Rape					–	–	1.284	–	–	–	–	–	–	–	–	–	–	–	
	Acacia					–	–	6.31	–	–	–	–	–	–	–	–	–	–	–	
5.	Knotweed		Banat	10	AAS mg/kg	–	0.114	0.954	–	0.220	–	–	–	–	–	–	–	–	–	[36]
	Linden					–	0.116	1.345	–	0.233	–	–	–	–	–	–	–	–	–	
	Acacia					–	0.114	0.902	–	0.249	–	–	–	–	–	–	–	–	–	
	Oilseed rape					–	0.110	4.999	–	0.199	–	–	–	–	–	–	–	–	–	
	Sunflower					–	0.108	0.551	–	0.202	–	–	–	–	–	–	–	–	–	
6.	Acacia		Bihor	6	ICP-OES mg/kg	–	0.030	1.103	–	0.046	–	–	–	–	–	–	–	–	–	[37]
	Honeydew			18		–	0.022	4.274	–	0.125	–	–	–	–	–	–	–	–	–	
	Heather			6		–	0.013	5.724	–	0.398	–	–	–	–	–	–	–	–	–	
	Lime			6		–	0.007	1.391	–	ND	–	–	–	–	–	–	–	–	–	
7.	Polyfloral		Baia Mare	–	FAAS mg/kg	–	–	–	–	–	–	–	–	–	–	–	–	–	–	[38]
8.	Multifloral	Italy	Lazio	40	ICP-OES μg/g	< 3-24	10-328	0.09-2.8	1.0-17	0.05-0.40	–	33-74	–	–	0.1-150	–	–	–	< 0.03-1.4	[39]
9.	Unifloral	Bulgaria	-	200	ICP-AES mg/kg	< 0.05	< 0.01-0.012	–	< 0.01	< 0.01-1.00	–	–	–	–	–	–	–	–	–	[40]
10.	Polyfloral	France	–	86	ICP-AES ppm	–	0.05-0.52	0.06-10.34	0.03-0.25	ND	–	–	–	0.08-2.16	–	–	–	–	–	[41]

11.	Fir	Greek	Messinia	207	ICP-OES mg/kg	–	0.02	3.74	–	0.38	–	0.18	–	< 0.01	0.01	–	–	–	0.01	[42]
12.	Lavender	Algeria	Sidi Djillali	37	ICP-MS (mg/kg)	0.009	0.07	13.30	0.009	–	–	–	–	–	–	–	–	–	–	[43]
	Rosemary					0.009	0.06	10.80	0.009	–	–	–	–	–	–	–	–	–	–	
	Multifloral					0.005	0.04	11.10	0.010	–	–	–	–	–	–	–	–	–	–	
13.	Acacia	Hungary	–	187	MP-AES (mg/kg)	–	–	3.3	–	–	–	–	–	–	–	–	–	–	–	[44]
	Honeydew					–	–	5.5	–	–	–	–	–	–	–	–	–	–	–	
	Forest					–	–	3.0	–	–	–	–	–	–	–	–	–	–	–	
	Sunflower					–	–	0.7	–	–	–	–	–	–	–	–	–	–	–	
	Chestnut					–	–	11.9	–	–	–	–	–	–	–	–	–	–	–	
	Rape					–	–	0.9	–	–	–	–	–	–	–	–	–	–	–	
	Multifloral					–	–	2.7	–	–	–	–	–	–	–	–	–	–	–	

Table S3. Heavy metals concentrations reported in various studies from honey.

No of Items	Denomination	Country	Location	No. of samples	Heavy metals						
					Method	^{75}As	^{111}Cd	^{201}Hg	^{208}Pb	^{238}U	Ref.
1.	Acacia	Romania	Domasnea	3	AAS (ppm)	–	ND	–	ND	–	[32]
			Farliug				ND		ND		
			Bala				ND		ND		
2.	Acacia, Lime, sunflower, and polyfloral		Dolj Mehedinți Gorj	–	AAS ppm	–	–	–	0.0030	–	[33]
3.	Polyfloral		Copșa Mică	–	FAAS GFAAS mg/kg	–	0.05-3.81	–	0.76-3.41	–	[34]
4.	Mixed Flowers P1		Timiș	–	AAS ppm	–	–	–	–	–	[35]
	Mixed Flowers P0					–	–	–	–	–	
	Linden P1					–	–	–	–	–	
	Linden P0					–	–	–	–	–	
	Rape					–	–	–	–	–	
	Acacia					–	–	–	–	–	
5.	Knotweed		Banat	10	AAS	–	0.130	–	0.163	–	[36]

	Linden				mg/kg	–	0.049	–	0.076	–	
	Acacia					–	0.078	–	0.109	–	
	Oilseed rape					–	0.099	–	0.118	–	
	Sunflower					–	0.061	–	0.131	–	
6.	Acacia		Bihor	6	ICP-OES mg/kg	–	ND	–	0.027	–	[37]
	Honeydew			18		–	ND	–	0.018	–	
	Heather			6		–	ND	–	0.031	–	
	Lime			6		–	ND	–	0.050	–	
7.	Polyfloral		Baia Mare	–	GFAAS mg/kg	–	ND-0.78	–	0.12-20.34	–	[38]
8.	Multifloral	Italy	Lazio	40	ICP-OES μg/g	< 25	1.3-4.2	–	9-209	0.04-1.0	[39]
9.	Unifloral	Bulgaria	–	200	ICP-AES mg/kg	< 0.1-0.268	< 0.1	–	< 0.08-0.31	–	[40]
10.	Polyfloral	France	-	86	ICP-AES ppm	–	ND	ND	ND	–	[41]
11.	Fir	Greek	Messinia	207	ICP-OES mg/kg	0.39	–	0.06	0.13	–	[42]
12.	Lavender	Algeria	Sidi Djillali	37	ICP-MS (mg/kg)	–	0.001	–	0.02	–	[43]
	Rosemary					0.005	0.0008	–	0.017	–	
	Multifloral					0.008	0.001	–	0.010	–	
13.	Acacia	Hungary	–	187	MP-AES (mg/kg)	–	–	–	0.5	–	[44]
	Honeydew					–	–	–	0.6	–	
	Forest					–	–	–	0.5	–	
	Sunflower					–	–	–	0.5	–	
	Chestnut					–	–	–	0.6	–	
	Rape					–	–	–	0.6	–	
	Multifloral					–	–	–	0.6	–	

Atomic absorption spectroscopy (AAS), flame atomic absorption spectrometry (FAAS), inductively coupled plasma-mass spectrometer (ICP-MS), inductively coupled plasma optical emission spectroscopy (ICP-OES), inductively coupled plasma atomic emission spectrometry (ICP-AES), microwave plasma atomic emission spectrometry (MP-AES), and graphite furnace atomic spectrometry (GFAAS).

Table S4. Descriptive attributes of honey samples, covering sample codes, specific qualities of honey, classification, geographical origins, harvest years, extraction methods, bee species, environmental factors, and anthropogenic influences.

Sample code	N° of sample	Harvest period	Honey details	Denomination	Geographical origin	Area/ Country	Year of harvest	Type of extraction	Environment	Anthropogenic influence	Bee species
H_{1-2020}	2	June	Certified origin	Multifloral	Galați	Romania	2020	Mechanical	Semi-rural	Near (~ 6.8 km distance) national highway (DN 25), with intense traffic of vehicles	*Apis mellifera*
H_{2-2021}	3	June	Certified origin	Linden	Galați	Romania	2021	Manual	Rural	Near (~ 1.5 km distance) national highway (DN 25), with intense traffic of vehicles	*Apis mellifera*
H_{3-2021}	1	June	Certified origin	Acacia	Galați	Romania	2021	Manual	Rural	Near (~ 3.1 km distance) national highway (DN 25), with intense traffic of vehicles	*Apis mellifera*
H_{4-2022}	2	June-July	Raw artisan honey	Sunflower	Târgu Bujor	Romania	2022	Mechanical	Rural	-	*Apis mellifera*
H_{5-2020}	2	June	Certified origin	Spring rape	Tecuci	Romania	2020	Mechanical	Semi-rural	Near (~ 800 m distance) country road (DJ 251), with intense traffic of vehicles	*Apis mellifera*
H_{6-2020}	1	May	Certified origin	Autumn rape	Tecuci	Romania	2020	Mechanical	Semi-rural	Near (~ 1000 m distance) country road (DJ 251), with intense traffic of vehicles	*Apis mellifera*
H_{7-2019}	2	June-July	Raw artisan honey	Sunflower	Vaslui	Romania	2019	Manual	Rural	Near (~ 6.5 km distance) national highway (DN 2F), with intense traffic of vehicles	*Apis mellifera*
H_{8-2018}	2	June	Raw artisan honey	Linden	Vaslui	Romania	2018	Manual	Semi-rural	Near (~ 6.5 km distance) national highway (DN 2F), with intense traffic of vehicles	*Apis mellifera*
H_{9}^{-2019}	3	June	Raw artisan honey	Lavender	Vaslui	Romania	2019	Manual	Rural	-	*Apis mellifera*
$H_{10-2021}$	3	June	Certified origin	Multifloral	Brăila	Romania	2021	Manual	Rural	Near (~ 12.0 km distance) European Road (E 87), with intense traffic of vehicles	*Apis mellifera*

$H_{11\text{-}2021}$	1	June	Raw artisan honey	Linden	Brăila	Romania	2022	Manual	Rural	Near (~ 12.0 km distance) European Road (E 87), with intense traffic of vehicles	*Apis mellifera*
$H_{12\text{-}2021}$	2	June-August	Raw artisan honey	Acacia + Linden	Brăila	Romania	2021	Manual	Rural	-	*Apis mellifera*
$H_{13\text{-}2021}$	1	June-August	Raw artisan honey	Multifloral	Brăila	Romania	2021	Manual	Rural	-	*Apis mellifera*
$H_{14\text{-}2020}$	1	May-June	Certified origin	Acacia	Satu Mare	Romania	2020	Manual	Rural	-	*Apis mellifera*
$H_{15\text{-}2020}$	2	June-August	Raw artisan honey	Multifloral	Satu Mare	Romania	2020	Manual	Rural	-	*Apis mellifera*
$H_{16\text{-}2020}$	1	June-August	Raw artisan honey	Multifloral	Tulcea	Romania	2020	Manual	Rural	-	*Apis mellifera*
$H_{17\text{-}2020}$	2	June-August	Raw artisan honey	Sunflower	Tulcea	Romania	2020	Manual	Rural	-	*Apis mellifera*
$H_{18\text{-}2019}$	1	June	Raw artisan honey	Linden	Tulcea	Romania	2019	Manual	Rural	-	*Apis mellifera*
$H_{19\text{-}2021}$	2	May-June	Certified origin	Honeydew	Botoșani	Romania	2021	Manual	Semi-rural	-	*Apis mellifera*
$H_{20\text{-}2021}$	3	June-August	Certified origin	Multifloral	Botoșani	Romania	2019	Manual	Semi-rural	-	*Apis mellifera*
$H_{21\text{-}2019}$	2	June-August	Certified origin	Linden	Botoșani	Romania	2019	Manual	Semi-rural	-	*Apis mellifera*
$H_{22\text{-}2019}$	1	June-August	Certified origin	Sunflower	Botoșani	Romania	2019	Manual	Semi-rural	-	*Apis mellifera*
$H_{23\text{-}2021}$	2	July-August	Certified origin	Acacia + Linden	Iași	Romania	2021	Mechanical	Rural	-	*Apis mellifera*

$H_{24\text{-}2020}$	2	June	Certified origin	Acacia	Iași	Romania	2020	Mechanical	Rural	-	*Apis mellifera*
$H_{25\text{-}2020}$	2	June	Certified origin	Sunflower	Iași	Romania	2020	Mechanical	Rural	-	*Apis mellifera*
$H_{26\text{-}2021}$	3	May-June	Raw artisan honey	Acacia	Sibiu	Romania	2021	Mechanical	Rural	Near (~ 14.0 km distance) European Road (E 81), with intense traffic of vehicles	*Apis mellifera*
$H_{27\text{-}2020}$	2	June-July	Raw artisan honey	Sunflower	Sibiu	Romania	2020	Mechanical	Rural	Near (~ 14.0 km distance) European Road (E 81), with intense traffic of vehicles	*Apis mellifera*
$H_{28\text{-}2019}$	1	June	Certified origin	Sunflower	Râmnicu Vâlcea	Romania	2019	Manual	Semi-rural	-	*Apis mellifera*
$H_{29\text{-}2018}$	1	June	Certified origin	Multifloral	Râmnicu Vâlcea	Romania	2018	Manual	Semi-rural	-	*Apis mellifera*
$H_{30\text{-}2018}$	2	May-June	Certified origin	Acacia	Arad	Romania	2018	Manual	Rural	Near (~ 21.3 km distance) European Road (E 68), with intense traffic of vehicles	*Apis mellifera*
$H_{31\text{-}2020}$	3	June	Certified origin	Sunflower	Teleorman	Romania	2020	Manual	Rural	-	*Apis mellifera*
$H_{32\text{-}2021}$	2	May-June	Certified origin	Acacia	Mehedinți	Romania	2021	Manual	Rural	Near (~ 7.5 km distance) national highway (DN 57), with intense traffic of vehicles	*Apis mellifera*
$H_{33\text{-}2021}$	1	June	Certified origin	Sunflower	Mehedinți	Romania	2021	Manual	Rural	Near (~ 7.5 km distance) national highway (DN 57), with intense traffic of vehicles	*Apis mellifera*

Table S5. The program of the microwave oven Milestone START D Microwave Digestion System

Step	Target Temp (°C)	Pressure Max. (psi)	Temperature Ramp (min.)	Hold Time (min.)	Power (%)
1.	220	800	10	20	100
2.	35-40	800	-	45 min. cooling	-

Table S6. Instrumental (a) and data acquisition (b) parameters of ICP-MS

(a) Instrumental parameters		(b) Data acquisition parameters for quantitative mode	
RF power/W	1.4 kW	Measuring mode	Standard (Ar 5.0) Q Cell (Collision Cell) (He 6.0)
Argon (Ar) gas flow, Helium (He) gas flow		Point per peak	3
Nebulizer	1.0 L/min.	Scans/Replicate	7
Plasma gas low rate (Ar 5.0)	18.0 L/min.	Replicate/Sample	7
Auxiliary gas flow rate (He 6.0)	0.20 L/min.		
Lens voltage	37 V	Dwell time (ms)	3
Mirror lens right	32 V		
Mirror lens bottom	31 V		
Sample uptake rate	90 s	Integration time	1-5 ms
Temperature spray chamber		2.10 °C	
Background correction		2 points/peak	
Injector tube		quartz 2-mm id	
Sample cone		Sample Cone 4450	
Skimmer cone		Ni – Skimmer iCAP Q 0.5 mm insert version	
Nebulizer		MicroMist Nebulizer 0.4 mL/min.	

Table S7. Instrumental conditions for the determination of each element using ICP-MS technique.

Element	Correlation coefficient	LoD (μg/L)	LoQ (μg/L)	BEC (μg/L)	Element	Correlation coefficient	LoD (μg/L)	LoQ (μg/L)	BEC (μg/L)
^{19}K	0.9991	2.847	7.321	31.733	^{23}Na	0.9996	3.991	13.232	32.121
^{24}Mg	0.9999	2.054	9.003	9.099	^{43}Ca	0.9995	5.384	17.986	21.004
^{7}Li	0.9992	0.005	0.032	0.020	^{27}Al	0.9992	0.068	0.324	6.006
^{56}Fe	0.9999	5.232	17.574	71.426	^{64}Cu	0.9997	0.035	0.139	0.236
^{65}Zn	0.9999	0.079	1.203	1.310	^{88}Sr	0.9997	0.133	0.476	0.957
^{9}Be	0.9999	0.006	0.020	0.015	^{51}V	0.9994	1.208	4.042	4.263
^{52}Cr	0.9999	1.607	5.533	0.637	^{55}Mn	0.9997	0.012	0.039	0.087
^{60}Ni	0.9997	0.045	0.181	0.096	^{70}Ga	0.9997	0.013	0.041	0.041
^{79}Se	0.9998	0.533	0.029	0.923	^{85}Rb	0.9996	0.151	0.230	0.653
^{204}Tl	0.9999	0.002	0.017	0.003	^{208}Ag	0.9993	0.018	0.166	0.017
^{209}Bi	0.9998	0.009	0.030	0.002	^{115}In	0.9997	0.004	0.011	0.009
^{133}Cs	0.9999	0.006	0.021	0.015	^{137}Ba	0.9998	0.879	0.169	2.684
^{75}As	0.9999	0.006	0.743	0.018	^{111}Cd	0.9997	0.007	0.069	0.0031
^{201}Hg	0.9999	0.043	0.137	0.128	^{208}Pb	0.9996	0.151	0.231	0.649
^{238}U	0.9999	0.031	0.084	0.005					

LoD = Detection limit; LoQ = Quantification limit; BEC = Background equivalent concentration.

Table S8. Validation parameters of the analytical procedure for the determination of each elements (honey)

Element	Certified reference material analysis		Validation parameters	
	The result declared by de manufacture	The results obtained in our research	Recovery (%)	Uncertainty (%)
$^{19}K^{b}$ (mg/kg)	0.107 ± 0.008	0.104 ± 0.001	99.13	20
$^{24}Mg^{a}$ (mg/kg)	4320 ± 150	4320 ± 132	99.82	16
^{7}Li (µg/L)	-	0.028	102.02	14
$^{56}Fe^{b}$ (mg/kg)	46 ± 2	45 ± 5	106.02	12
$^{59}Co^{d}$ (mg/kg)	0.5773 ± 0.071	0.6541 ± 0.321	107.18	24
$^{65}Zn^{b}$ (mg/kg)	38 ± 2	31.84 ± 0.86	100.12	17
^{9}Be (µg/L)	-	0.023	99.04	23
$^{52}Cr^{a}$ (mg/kg) (not certified)	1.988 ± 0.034	2.124 ± 0.070	92.69	21
$^{60}Ni^{a}$ (mg/kg)	0.689 ± 0.095	1.784 ± 0.084	98.70	18
$^{79}Se^{d}$ (mg/kg)	0.0543 ± 0.0020	0.0513 ± 0.0018	105.22	14
^{204}Tl (µg/L)	-	0.023	99.85	16
^{209}Bi (µg/L)	-	0.014	113.12	22
$^{133}Cs^{d}$ (mg/kg) (not certified)	0.053	0.046 ± 0.087	98.98	19
$^{75}As^{a}$ (mg/kg)	0.062 ± 0.014	0.1126 ± 0.062	117.89	16
$^{201}Hg^{b}$ (mg/kg)	0.0399 ± 0.0007	0.023 ± 0.017	95.84	10
$^{238}U^{d}$ (mg/kg) (not certified)	0.035	0.025 ± 0.001	112.09	9
$^{23}Na^{c}$ mg/kg (not certified)	24.4 ± 2.1	23.4 ± 3.5	98.99	14
$^{43}Ca^{b}$ (mg/kg)	0.25 ± 0.01	0.26 ± 2.9	111.87	13
$^{27}Al^{b}$ (mg/kg)	580 ± 30	567.89 ± 10	99.98	23
$^{64}Cu^{b}$ (mg/kg)	2.8 ± 0.2	4.42 ± 0.31	96.87	23
$^{88}Sr^{a}$ (mg/kg)	53 ± 5.0	51 ± 1.1	110.21	13
$^{51}V^{a}$ (mg/kg)	0.367 ± 0.038	0.389 ± 0.024	98.87	16
$^{55}Mn^{a}$ (mg/kg)	97.8 ± 1.8	99.1 ± 2.4	99.00	22
^{70}Ga (µg/L)	-	0.044	99.89	13
$^{85}Rb^{b}$ (mg/kg)	16.5 ± 0.9	15.3 ± 1.8	111.01	20
$^{208}Ag^{d}$ (mg/kg) (not certified)	0.017	0.013 ± 0.001	101.89	12
^{115}In (µg/L)	-	0.009	111.45	21
$^{137}Ba^{b}$ (mg/kg)	6.0 ± 0.2	5.98 ± 1.2	98.97	18
$^{111}Cd^{b}$ (mg/kg)	0.233 ± 0.004	1.687 ± 0.147	90.32	23
$^{208}Pb^{a}$(mg/kg)	0.869 ± 0.018	0.164 ± 0.003	96.78	22

[a] NIST – 1547 Peach Leaves Standard Reference Materials; [b] NIST – 1575a Pine Needles (*Pinus taeda*) Standard Reference Materials; [c] NIST – 1515 Apples Leaves Standard Reference Materials; [d] NIST – 1573a Tomato Leves Standard Reference Materials.

Table S9. Quantitative expressions denoting the proportion of each individual elements in terms of percentage values (%).

Element	Percentage Calculation %
^{19}K	84.04
^{23}Na	2.16
^{24}Mg	3.86
^{43}Ca	8.05
^{7}Li	0.02
^{27}Al	0
^{56}Fe	1.52
^{64}Cu	0.06
^{65}Zn	0.07
^{88}Sr	0.01
^{9}Be	0
^{51}V	0
^{52}Cr	0.06
^{55}Mn	0.15
^{59}Co	0
^{60}Ni	0.01
^{70}Ga	0
^{79}Se	0
^{85}Rb	0
^{204}Tl	0
^{208}Ag	0
^{209}Bi	0
^{115}In	0
^{133}Cs	0
^{137}Ba	0
^{75}As	0
^{111}Cd	0.03
^{201}Hg	0
^{208}Pb	0.09
^{238}U	0
Σ	100
